# Effectiveness of a Web-Based Self-Guided Intervention (MINDxYOU) for Reducing Stress and Promoting Mental Health Among Health Professionals: Results From a Stepped-Wedge Cluster Randomized Trial

**DOI:** 10.2196/59653

**Published:** 2025-02-03

**Authors:** Yolanda López-del-Hoyo, Selene Fernández-Martínez, Adrian Perez-Aranda, Alicia Monreal-Bartolomé, Alberto Barceló-Soler, Loreto Camarero-Grados, Carilene Armas-Landaeta, José Guzmán-Parra, Vera Carbonell, Daniel Campos, Xinyuan Chen, Javier García-Campayo

**Affiliations:** 1 Faculty of Social Sciences University of Zaragoza Zaragoza Spain; 2 Institute of Health Research of Aragon Zaragoza Spain; 3 Research Network on Chronicity, Primary Care and Health Promotion (RICAPPS) Zaragoza Spain; 4 Faculty of Medicine University of Zaragoza Zaragoza Spain; 5 Department of Clinical and Health Psychology Universitat Autònoma de Barcelona Bellaterra, Barcelona Spain; 6 CIBER of Epidemiology and Public Health (CIBERESP) Madrid Spain; 7 Department of Psychology and Sociology Faculty of Education University of Zaragoza Zaragoza Spain; 8 Instituto de Investigación Biomedicina de Málaga Málaga Spain; 9 Unidad de Gestión Clínica de Salud Mental Hospital Regional Universitario de Málaga Málaga Spain; 10 Department of Mathematics and Statistics Mississippi State University, MS United States

**Keywords:** eHealth, health professionals, stress, mental health, stress reduction, web-based intervention, stepped-wedge design, randomized controlled trial

## Abstract

**Background:**

The high levels of sustained stress that health professionals often experience are a significant risk factor for developing mental health problems, such as anxiety, depression, and somatic symptoms, that not only affect their well-being but also have major social and organizational consequences. Different interventions, including those based on third-wave psychotherapy principles (ie, mindfulness, compassion, and acceptance), have proven to be effective in reducing stress in this population. Among them, those delivered on the web constitute a promising alternative with notable advantages in accessibility and flexibility, but some adherence inconveniences may limit their efficacy.

**Objective:**

This study aimed to evaluate the effectiveness of the MINDxYOU program, a web-based self-guided intervention based on third-wave psychotherapy principles, to reduce perceived stress and promote mental health in a sample of health professionals.

**Methods:**

In a stepped-wedge cluster randomized design, 357 health professionals from health centers in Aragon and Málaga, Spain, were recruited. They were divided into 6 clusters—3 per region—and randomly assigned to 1 of the 3 sequences, each starting with a control phase and then transitioning to the intervention phase (the MINDxYOU program) after 8, 16, or 24 weeks. This self-guided, web-based program, designed to be completed over 8 weeks, included weekly contact (via WhatsApp, call, or email) from the research team to promote adherence. Participants were assessed on the web every 8 weeks for 5 assessments. Perceived stress was the study’s primary outcome, with additional measures of clinical factors (anxiety, depression, and somatization) and process variables (resilience, mindfulness, compassion, and acceptance).

**Results:**

The program was initiated by 229 participants, 112 (48.9%) of whom were completers (ie, completed at least 3 of the 4 modules). Perceived stress demonstrated a significant reduction both when considering the entire sample (β=–1.08, SE 0.51; *P*=.03) and the sample of completers (β=–1.84, SE 0.62; *P*=.003). The proportion of participants reflecting “low stress” increased after the treatment (n=90, 46.6% vs n=100, 28.8% at baseline). Intracluster analysis revealed that pre- versus postintervention moderate effects were present in 2 clusters (Cohen *d*=0.46 and 0.62), and these were maintained in subsequent assessments. The linear mixed-effects models also showed that depression, anxiety, and somatization, as well as resilience, self-compassion, and some mindfulness facets, experienced significant improvements (*P*<.05) when comparing the intervention and control phases.

**Conclusions:**

The MINDxYOU program was effective in reducing perceived stress and promoting mental health, as well as increasing resilience, mindfulness facets, and self-compassion. These effects suggest that participants experienced a tangible improvement that could potentially enhance their well-being. Adherence to the intervention was moderate, while program use was notable compared to similar interventions. Finding ways to promote adherence to the intervention would contribute to increasing the effectiveness of this program.

**Trial Registration:**

ClinicalTrials.gov NCT05436717; https://clinicaltrials.gov/study/NCT05436717

**International Registered Report Identifier (IRRID):**

RR2-10.1186/s12912-022-01089-5

## Introduction

### Background

Health professionals often experience high levels of sustained stress that constitute a risk for developing mental health problems, as recognized by the World Health Organization [1,2]. In recent years, the prevalence of psychological disorders such as anxiety, depression, and somatic symptoms has increased among this population, which has been linked to the extra efforts exerted by health workers and the extremely difficult situations to which they were exposed during the COVID-19 pandemic [3-6]. Although the pandemic was declared officially over in May 2023, its effects on the well-being of health professionals have been observed to be profound and lasting in many cases [7,8]. The implications of mental health problems in these professionals not only refer to their well-being but also include social and organizational consequences, such as increasing rates of absenteeism and turnover intention [9-11], as well as so-called quiet quitting [12]. Thus, despite the end of the COVID-19 pandemic, it is essential to continue to monitor health professionals’ mental health and implement effective strategies to decrease their stress levels, thus preventing chronic stress and its consequences [13].

The effectiveness of interventions based on cognitive behavioral therapy (CBT) on stress reduction and mental health promotion has been widely proven for different types of health professionals [14-16]. Relaxation, cognitive restructuring, and social skill training, among others, are traditional CBT techniques aimed at modifying the interaction between thoughts, behaviors, and emotions. However, in recent decades, a different approach to CBT has been developed and tested with positive results. Referred to as the third wave of CBT psychotherapy, it is based on the promotion of mindfulness, acceptance, compassion, and spirituality [17]. Different systematic reviews have underscored the effectiveness of third-wave psychotherapies, including mindfulness-based interventions, compassion-based programs, and acceptance and commitment therapy, in reducing occupational stress as well as anxiety and depression while increasing resilience, including in health professionals [18-21].

While psychological interventions are typically delivered in an in-person format, eHealth interventions—examples of which are telehealth, mobile apps, and serious games—have proliferated over the last few years, partly due to the COVID-19 pandemic [22]. The systematic review conducted by López-Del-Hoyo et al [23] summarized the different types of eHealth programs that have been tested to promote mental health in health professionals and concluded that there was promising evidence of the effectiveness of self-guided, web-based, third-wave psychotherapy–based programs for reducing stress and promoting mental health. The self-guided format, which implies the lack of an external figure who delivers the contents of the intervention, offers a high degree of availability, flexibility, and convenience [24]. This could be particularly useful in the case of health professionals who often have to cope with the difficulty of reconciling work schedules with attending therapy sessions [25]. Indeed, this type of eHealth program has been associated with high user satisfaction and acceptance [26,27].

Nonetheless, some self-guided interventions have turned out to be ineffective for health professionals, and notably low adherence rates to these programs have been observed [23]. This may be due to generic and unappealing programs not addressing the particular needs of these individuals, although it has also been suggested that their lack of external guidance could contribute to higher attrition rates [26,28]. This would imply that the flexible and adaptable nature of self-guided programs can be both an advantage and an obstacle while achieving significant improvements in stress and other mental health–related outcomes. Therefore, a mixed format, that is, a self-guided web-based program that includes occasional—preferably, remote and asynchronous—contact with an external figure, via WhatsApp messages or emails, for instance, could present a solution for retaining the main advantages of self-guided web-based programs while reducing the risk of attrition [23].

### Objectives

The aim of this study was to test the effectiveness of a self-guided web-based program (MINDxYOU) for reducing the perceived stress of health professionals while promoting different mental health–related outcomes through the application of third-wave psychotherapy principles: mindfulness, compassion, and acceptance. Following the recommendations of previous authors [29], the MINDxYOU program was not designed as a generic program to be used by any given population; rather, its specific contents and examples relate to the day-to-day activities of health professionals (eg, compassion fatigue and common stressors). The program consists of 8 parts (sessions) to be completed within 8 consecutive weeks. During this time, weekly contact was scheduled with the research team via email, WhatsApp, or phone call to include a small degree of guidance in the intervention. The MINDxYOU program highlights the importance of systematic and continuous practicing of different types of meditation not only during the 8-week duration of the intervention but also afterward to promote the development of new healthy habits. It was hypothesized that on completing the web-based program, health professionals would experience a significant reduction in their stress levels as well as improvements in other clinical aspects (anxiety, depression, and somatization) and process variables (resilience, mindfulness, compassion, and acceptance).

## Methods

### Study Design

A stepped-wedge cluster randomized trial design [30] was used for this hybrid type 2, effectiveness and implementation study, which was conducted in 2 Spanish regions (the autonomous region of Aragon and the province of Malaga, in Andalusia). This design was chosen considering its suitability for studying both the effectiveness of the MINDxYOU program (reported in this work) and its implementation in health centers (to be reported in a different work) [31,32]. The stepped-wedge design allows all the study participants to receive the intervention and is particularly recommended when the intervention being tested has strong evidence of positive effects or is very unlikely to cause harm [30].

The participants were health professionals working for different types of health care institutions: hospitals, primary care centers, and other health facilities (eg, nursing homes and health and social care centers). The sample was divided into 6 clusters—3 per region—which were randomly allocated to 1 of the 3 possible sequences. They all started at the control phase and sequentially transitioned to the intervention phase (they were given access to the MINDxYOU program). This was a closed cohort study, given that all the participants started at the same time and were evaluated at 5 previously defined time points.

As detailed in the study protocol [33], the sample size calculation estimated 180 participants, 30 (16.7%) per cluster, for an expected moderate effect size of the intervention on the primary outcome, that is, perceived stress [34]. However, the ambitious dissemination of the project attracted the interest of a very high number of health professionals, and an effort was subsequently made to recruit 393 participants, of whom 357 (90.8%) would ultimately provide data and consequently form the study sample.

### Recruitment and Inclusion Criteria

Between October 2022 and January 2023, the MINDxYOU project was disseminated using both direct and indirect techniques. Informative meetings were conducted for managers and health professionals working in health facilities (hospitals, primary care centers, and nursing homes) in Aragon and Malaga; posters and leaflets were placed at different health centers; and members of the research group were interviewed by regional media regarding the project, optimizing its visibility. Individuals interested in participating accessed the project’s website [35] to complete a short form to verify whether they met the inclusion criteria as follows: (1) employment as a health professional (eg, physician, nurse, psychologist, or nursing assistant) or undergoing training in any health-related field (trainee); (2) aged between 18 and 70 years; (3) ability to understand Spanish; (4) digital literacy and access to a smartphone, tablet, or PC with an internet connection; and (5) prospects of continued employment at the same workplace for the following 6 months. Candidates who met these criteria were also asked on the form to provide their email address or telephone number for subsequent contact by the researchers.

Every candidate underwent a telephone interview conducted by a member of the research team, either a psychologist or mental health nurse trained for this purpose, to ensure the previously described inclusion criteria were met. In addition, the following exclusion criteria were assessed during the interview: (1) presenting with a disorder affecting the central nervous system; (2) diagnosis of a severe mental illness (severe depressive disorders, suicidal tendencies, bipolar disorders, panic disorders, anxiety or stress-related disorders, obsessive-compulsive disorders, and substance-related disorders) using the Mini-International Neuropsychiatric Interview (version 7.0.2) diagnostic interview [36]; (3) presenting with an uncontrolled medical condition or either an infectious or degenerative disease; and (4) having experience with third-wave psychotherapies (eg, having attended mindfulness courses in the previous year or practicing formal meditation regularly). Candidates who fit the inclusion profile were sent an email containing the study information and an attached informed consent form to be signed electronically and returned.

### Procedures

Once the participants had been enrolled in the study (February 2023), they were assigned a code to access the evaluation platform (SurveyMonkey [SurveyMonkey Inc]) and ensure their anonymity throughout the study. An external researcher unrelated to this project then performed the cluster randomization under 2 conditions: each sequence had to include a cluster from each region, and they had to be 2 different types of workplaces (hospitals, primary care centers, or other health centers). The baseline assessment took place over the last week of February 2023 and the first week of March 2023. Following the stepped-wedge design, this assessment was repeated every 8 weeks, which was the expected duration of the program. There were 5 assessment points in total.

Emails were sent to all participants after each assessment to indicate whether they had been granted access to the MINDxYOU program or still had to wait a certain amount of time (ie, they remained in the control phase). Once they were allowed access, they first watched a 3-minute video tutorial on how to log into the platform and use it. The introduction to the program (module 0) explained to the participants the format of the intervention (4 modules, each containing 2 sessions). Although visualizing the contents of each session (text and videos) would take approximately 1 hour, it was recommended that participants dedicate an entire week to each session before moving on to the next, because every session included meditation exercises that needed to be practiced on consecutive days to be learned and incorporated into a routine if they were to produce stress-relief effects. Therefore, the program was expected to be completed in 8 weeks (1 session per week). Whenever participants indicated their intention to start a new session, the platform would present a brief on the web form asking how many days they had been practicing the exercises from the previous session. This was meant to remind them of the recommendation to dedicate 1 full week to the series of exercises in each session before moving on to the next one. Nevertheless, all participants were given access to the following sessions when they completed the form, regardless of their dedication to the previous session.

Once per week during the 8-week duration of the program, all (N=357, 100%) participants were contacted via their preferred means (email, WhatsApp, or phone call, as indicated when they signed the informed consent) by a member of the research team (SF-M, CA-L, LC-G, or VC). The purpose of this contact was to motivate the participants to adhere to the intervention as per the recommendations and identify any difficulties with the program regarding its contents or use of the platform. If any of these were communicated by participants, the researcher would provide help to clarify and resolve the issue. The weekly contacts ended after the eighth week, regardless of the degree to which each participant had completed the program by that time. Nonetheless, the participants were informed that they could continue to use the MINDxYOU program and were encouraged to keep practicing what they had learned. Follow-up emails were sent reminding every study participant of this information.

### Intervention

The MINDxYOU program is a self-guided web-based intervention delivered via a digital platform that consists of 4 modules, each of them divided into 2 parts (sessions). The contents of the intervention are related to third-wave psychotherapies (mindfulness, compassion, and acceptance), which have presented strong evidence of efficacy for reducing stress and promoting mental health [18]. All the contents of the MINDxYOU program are based on previously developed and validated programs, such as mindfulness-based stress reduction [37], Smiling is Fun [38], and attachment-based compassion therapy [39]. Following the suggestions of previous studies [23,29], the MINDxYOU program includes a series of adaptations to make it more specific to health professionals (eg, examples of typical stressors that occur in health care settings). Both formal and informal practices are presented during the program: downloadable audio guides are provided for formal practice, while informal exercises are linked to the promotion of healthy habits, such as physical activity, healthy diet, and good sleep habits, in addition to socializing in module 4. The intervention emphasizes the importance of including meditation exercises in a daily routine and developing healthy habits that can have a stress-relief effect. Consequently, participants are encouraged to download the materials and continue to practice even after the completion of the program. A summary of the contents and exercises proposed for each session is presented in Multimedia Appendix 1.

### Measurements

#### Overview

Sociodemographic questions (age, gender, marital status, and education level) and work-related questions (type of contract, occupation, management position, trainee status, and salary) were presented to the participants at baseline assessment. These questions were not presented again at subsequent assessments, although participants were asked whether they still worked for the same health care institution (and if not, the one at which they were currently employed). In addition to these questions, the primary and secondary outcomes and process variables were included at every assessment point.

#### Primary Outcome

The Perceived Stress Scale (PSS) [40] was the primary outcome of this study. It is a 10-item questionnaire that measures how unpredictable, uncontrollable, and overloaded the individual has felt their life to be over the previous month on a 5-point Likert-type scale. Scores range between 0 and 40, with higher scores reflecting higher levels of perceived stress. Cutoff points have been established to determine 3 categories of stress levels: low (0-13), moderate (14-26), and high (27-40). Our study made use of the Spanish adaptation [41], and the PSS for our sample showed very high internal consistency (α_T1_=0.89 and α_T2-T5_=0.91).

#### Secondary Outcomes

The Patient Health Questionnaire-9 (PHQ-9) [42] is a 9-item scale that rates the frequency of depressive symptoms during the previous 2 weeks using a Likert-type scale (from 0=not at all to 3=nearly every day). The total score ranges between 0 and 27, with higher scores indicating higher severity of depression. The Spanish adaptation of the PHQ-9 was used for this study [43], and we observed good internal consistency in our sample (α_T1_=0.83, α_T2_=0.88, α_T3_=0.86, α_T4_=0.89, and α_T5_=0.87).

The Generalized Anxiety Disorder-7 (GAD-7) [44] is a 7-item, self-report measure to assess the intensity of anxiety symptoms over the past 2 weeks. Using a 4-point Likert-type scale, the total score can range between 0 and 21, with higher values indicating more severe anxiety symptoms. The Spanish version of the GAD-7 [45] was used for this study, and excellent psychometric properties were observed in our sample (α_T1_=0.89, α_T2_=0.92, α_T3_=0.90, α_T4_=0.92, and α_T5_=0.91).

The Brief Symptoms Inventory-18 (BSI-18) [46,47] is a 5-point Likert-type scale designed to offer rapid screening for the symptoms of psychological disorders (somatization, depression, and anxiety). Scores on the 18 items are summarized on the Global Severity Index, which ranges from 0 to 72, with higher scores reflecting more severe conditions. The Spanish version of the BSI-18 was used [48] and showed excellent internal consistency for our sample (α_T1_=0.90, α_T2-T3_=0.92, α_T4_=0.94, and α_T5_=0.92).

#### Process Variables

In total, 5 process variables were evaluated at each assessment point: resilience, mindfulness, self-compassion, compassion for others, and acceptance. Resilience was measured using the Connor-Davidson Resilience Scale (CD-RISC) [49], a 10-item scale whose total score ranges between 0 and 40, with higher scores indicating higher levels of resilience. Mindfulness was assessed using the Five Facet Mindfulness Questionnaire-15 (FFMQ-15) [50], which includes 5 subscales: observing, describing, acting with awareness, nonjudging of inner experience, and not reacting to inner experience. Each FFMQ-15 subscale includes 3 items, scored on a Likert-type scale (1-5), and the average score is calculated (higher scores indicate higher levels of the mindfulness facet). Both self-compassion and compassion for others were assessed using the Sussex-Oxford Compassion Scales (SOCS) [51]; each scale consists of 20 items, and its score is calculated by adding the scores of its items (ranging between 20 and 100, with higher scores indicating higher levels of compassion). Finally, the Acceptance and Action Questionnaire-II (AAQ-II) [52] was used to evaluate acceptance as opposed to experiential avoidance. The scale presents 7 items scored on a 7-point Likert-type scale, and the total score ranges between 7 and 49, with higher scores indicating higher experiential avoidance (ie, lower acceptance).

For this study, the Spanish version of the CD-RISC [53], FFMQ-15 [54], SOCS [55], and AAQ-II [56] were used. All showed good or excellent psychometric properties for our sample (α≥0.80 at every assessment point), except for FFMQ-observing (α_T1_=0.74, α_T2_=0.55, α_T3_=0.70, and α_T4-T5_=0.74), FFMQ-describing (α_T1,T2, and T5_=0.77), and FFMQ-nonreacting (α_T1_=0.71, α_T2-T3_=0.58, α_T4_=0.60, and α_T5_=0.71).

#### Adherence to the MINDxYOU Program

Adherence to the intervention was only determined by assessments completed after the participants had been given access to the MINDxYOU program, in which they were asked to state (1) the number of modules they had completed (where they had previously completed the intervention, they were asked how often they had revisited the program in the previous 2 months), (2) an average number of days per week they had engaged with formal practice (including the exercises they performed and their duration), and (3) an average number of days per week they had engaged with informal practice (including the exercises they had performed). In addition, every time participants wanted to access a new session while on the program, they had to complete a brief form indicating how many days they had practiced the exercises that were presented in the previous session.

### Data Analysis

First, we describe participant flow and compliance with the intervention. Frequencies and percentages were used to summarize the number of participants who completed all assessments, initiated the program, and completed at least 3 of the 4 modules. The chi-square test or ANOVA tests were conducted to assess between‐cluster differences as well as potential differences between (1) participants who dropped out and those who completed all the assessments, (2) participants who ultimately initiated the program and those who did not, and (3) the individuals who were considered to have completed the program and all the others.

Sociodemographic and clinical baseline data as well as intervention adherence-related data were described using means and SDs for continuous variables and frequencies and percentages for categorical variables. Chi-square tests and ANOVA were conducted to assess between-cluster differences in baseline characteristics.

The primary analysis (the effects of the intervention on perceived stress) was performed using linear mixed-effects models with cluster-specific random effects and time-specific fixed effects. We adjusted the model to include the following baseline imbalanced covariates (covariate-adjusted analysis): cluster, age, education level, type of contract, occupation, trainee status, salary, and the baseline level of the outcome. A cluster-robust sandwich variance estimator with small-sample corrections was implemented to obtain bias-corrected SEs [57]. We report the effect estimate, SE, and 95% CI. These analyses were conducted following 2 different approaches: the intention-to-treat approach (all the study participants included) and the per-protocol approach (program noncompleters excluded in postintervention assessments). The same strategy was used to analyze the effects on secondary outcomes and process variables. In addition, for the primary outcome, intracluster analyses under both approaches (intention-to-treat and per-protocol approaches) were computed using 2-tailed Student *t* test, whereas Cohen *d* was used to estimate the effect size.

An α level of .05 was set, using a 2‐tailed test. For the primary analysis, a completely missing at random approach was chosen given the characteristics of the study design and the sample size. For the intracluster analyses, we initially used raw data; however, we later performed a simple imputation (mean of nearby points) to assess potential effects under both the intention-to-treat and per-protocol. We applied the Benjamini-Hochberg correction for multiple comparisons in every case other than the primary analysis, following the recommendations by Li et al [58]. This is a procedure to detect false discovery designed to overcome the limitations that the other tests typically used have shown [59,60]. Data analyses were computed using SPSS (version 26.0; IBM Corp) and R (R Foundation for Statistical Computing) statistical software.

### Ethical Considerations

The research ethics committee of the Autonomous Community of Aragon and the ethics and research committee of Northeast Malaga evaluated and approved the study protocol in July 2022 (PI22/341). All procedures performed in this study adhered to the 1964 Declaration of Helsinki and its most recent amendments (seventh revision, adopted by the 64th World Medical Association General Assembly, Fortaleza, Brazil). A signed informed consent was obtained from all participants after they were informed of the study procedures, potential risks, and their right to withdraw at any time from the study. The participants did not receive any compensation. The study ensured the confidentiality of the information collected by fully complying with the Spanish Data Protection and Digital Rights Act 3/2018 and the European Union’s General Data Protection Regulation (GDPR). To maximize data protection, the technological infrastructure consisted of two independent systems accessing separate databases, ensuring the complete disaggregation of users’ personal data from their clinical records. This study was registered in ClinicalTrials.gov on June 29, 2022 (NCT05436717), and this work follows the CONSORT (Consolidated Standards of Reporting Trials) extension for the stepped-wedge cluster randomized trial [61] and the CONSORT-eHEALTH checklist [62].

## Results

### Flow and Compliance

**Figure 1:**
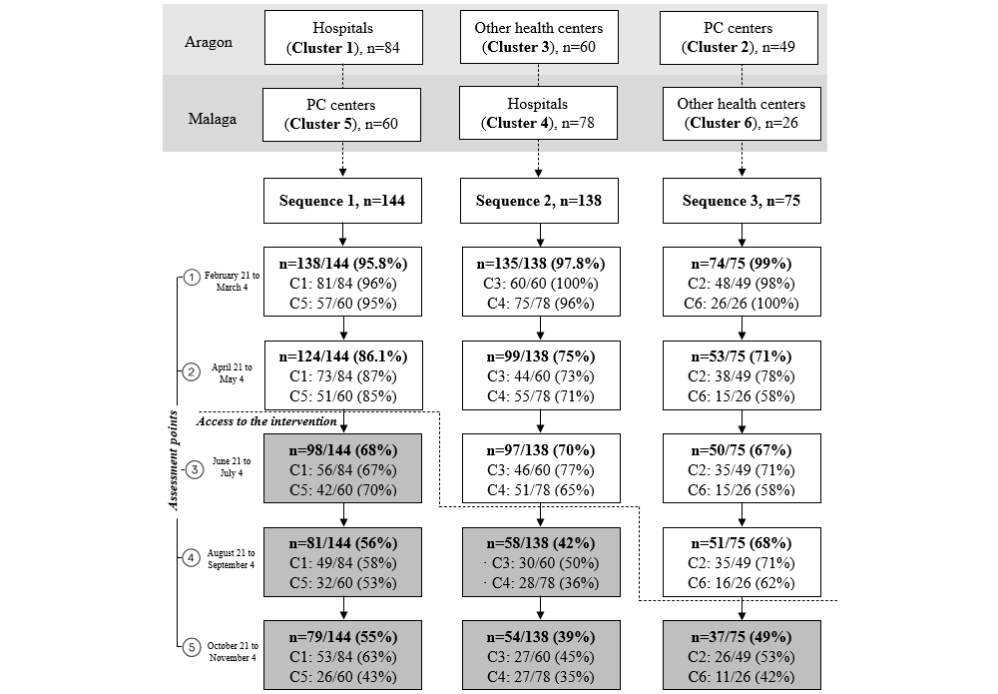
Study flowchart. PC: primary care. C1-6: cluster 1-6.

In total, 393 participants were included in the study, although 36 (9.2%) of them did not complete any assessment. Therefore, the study sample comprised 357 participants (Figure 1 for cluster distribution). Some significant differences (*P*<.05) were found between individuals who completed all the assessments (n=131, 36.7%) and those who did not (n=226, 63.3%; Multimedia Appendix 2). Participants in clusters 4 and 6 had a higher rate of assessment incompletion (*χ*^2^*_5_*=14.5; *P*=.001), as did those from Malaga (*χ*^2^*_1_*=9.8; *P*=.002).

As shown in Table 1, the MINDxYOU program was initiated by 64.1% (229/357) of the participants. After applying the Benjamini-Hochberg correction, a significantly higher proportion of participants who did not initiate the program was observed among those coming from Malaga (*χ*^2^*_1_*=9.9; *P*=.002) and clusters 4 and 6 (*χ*^2^*_5_*=19.9; *P*=.001; Multimedia Appendix 3). With regard to program completion, 48.9% (112/229 initiators) of the participants completed at least 3 modules, and 43.7% (100/229) completed all of them. Here, no significant differences were found when comparing completers with noncompleters (Multimedia Appendix 4). On average, initiators of the program completed 2.2 (SD 1.8) modules.

**Table 1:** Completion of the MINDxYOU program.

	Started the program, n (%)	Completed module 1, n (%)	Completed module 2, n (%)	Completed module 3, n (%)	Completed all 4 modules, n (%)
Cluster 1 (n=84)	65 (77)	45 (54)	42 (50)	35 (42)	31 (37)
Cluster 2 (n=49)	31 (63)	23 (47)	19 (39)	16 (33)	15 (31)
Cluster 3 (n=60)	42 (70)	29 (48)	24 (40)	21 (35)	18 (30)
Cluster 4 (n=78)	36 (46)	26 (33)	19 (24)	16 (21)	14 (18)
Cluster 5 (n=60)	41 (68)	26 (43)	20 (33)	20 (33)	18 (30)
Cluster 6 (n=26)	14 (54)	10 (38)	6 (23)	4 (15)	4 (15)
Total (N=357)	229 (64.1)	159 (44.5)	130 (36.4)	112 (31.4)	100 (28)

The 229 participants who initiated the program dedicated an average of 4.3 (SD 2.6) days to each session. After the intervention, participants (n=148) reported an average formal practice frequency of 3.3 (SD 1.5) days per week, with a mean duration of 18.8 (SD 11.6) minutes per exercise. Informal exercises were performed an average of 3.5 (SD 2) days per week. In the second postintervention assessment (clusters 1, 3, 4, and 5), participants (n=94) reported a formal practice frequency of 2.5 (SD 1.4) days per week, 15.25 (SD 10.2) minutes per exercise, and 3.3 (SD 1.9) days per week of informal practice. In the third postintervention evaluation (clusters 1 and 5, n=59), the frequency of formal practice remained at 2.6 (SD 1.7) days per week, 13.8 (SD 11.3) minutes per exercise, and 3.5 (SD 2.2) days per week of informal practice. No significant differences between clusters were observed in any case (*P*>.05 in all cases).

### Baseline Characteristics

As shown in Multimedia Appendix 5, the sample comprised mostly women (297/347, 85.6%) who were middle aged (mean 45.01, SD 11.17) and married (260/347, 74.9%), and most had completed university-level studies (304/347, 87.6%). Some between-cluster differences were observed. Cluster 6 was younger (*F*_5_=5.66; *P*<.001), and cluster 3 presented a lower education level (*χ*^2^_10_=68.8; *P*<.001).

Most participants were public-sector employees on permanent contracts (159/347, 45.8%), and the most common professions in our sample were physicians (147/347, 42.4%) and nurses (93/347, 26.8%). Only 15% (52/347) of participants occupied a management position at their institution, and 9.8% (34/347) were trainees for some health profession. Most (146/347, 42.1%) participants had a salary equivalent to between 1 and 2 times the minimum wage (between €1000 and €2000 per month; €1=US $1.04). Again, some between-cluster differences were observed. Cluster 6 had a very low number of functionaries (*χ*^2^_5_=97.3; *P*<.001) and physicians (*χ*^2^_25_=131.2; *P*<.001) compared to the other clusters; trainees were not represented in clusters 3 and 6 (*χ*^2^_5_=15.6; *P*=.008), and these clusters had a lower salary (*χ*^2^_15_=62.3; *P*<.001).

With regard to the clinical variables at baseline (Multimedia Appendix 5), our sample presented moderate levels of perceived stress, as indicated by the mean score in the PSS (mean 16.88, SD 6.33) and the fact that most (225/347, 64.8%) participants presented scores that fell under the moderate stress category (ie, 14-26 points of 40). Low levels of depression (PHQ-9: mean 6.25, SD 4.36), anxiety (GAD-7: mean 7.05, SD 4.23), and psychopathological symptoms (BSI-18: mean 12.34, SD 9.88) were observed. Participants showed a notable degree of resilience (CD-RISC: mean 27.31, SD 6.79), moderate levels of mindfulness facets (FFMQ-15 scores around 3), lower levels of self-compassion (SOCS-self: mean 53.53, SD 10.24) than compassion for others (SOCS-Others: mean 61.90, SD 8.77), and moderate-low levels of experiential avoidance, that is, moderate-high levels of acceptance (AAQ-II: mean 20.89, SD 8.33). Self-compassion (SOCS-self) was the only variable that showed significant between-cluster differences after applying the Benjamini-Hochberg correction (*F*_5_=3.20; *P*=.008).

### Effects on Perceived Stress

**Figure 2:**
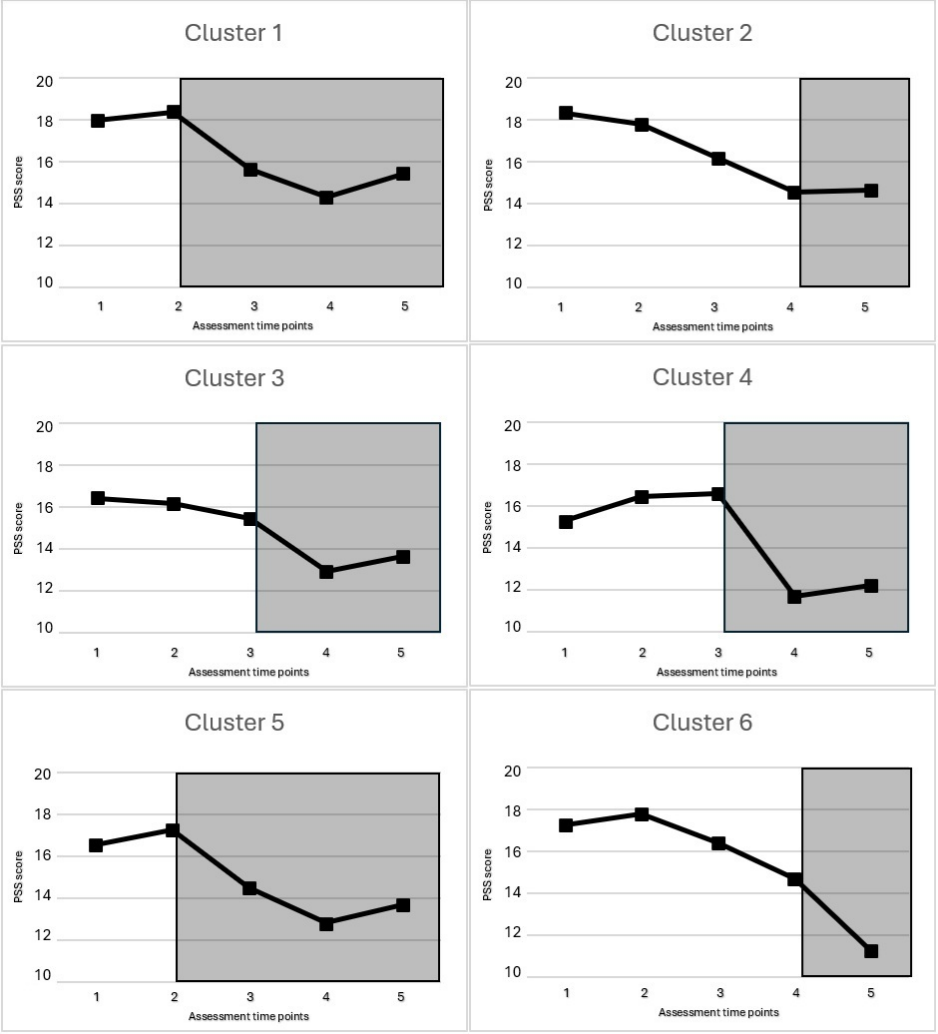
The evolution of Perceived Stress Scale scores across the study assessments per cluster. Squares in grey (transparent) indicate the periods in which the participants had access to the intervention.

PSS preintervention scores were 16.63 (SD 6.89) and were reduced to 14.02 (SD 6.69) after the intervention. The change was significant under both analytical approaches (Table 2), although stronger under the per-protocol approach. Some of the covariates included in the model were found to be significant: the sequence in which the cluster was allocated, type of contract, occupation, salary, and baseline PSS level. While at baseline most participants referred to moderate stress (225/347, 64.8%) and fewer reported low stress (100/347, 28.8%), after the treatment these proportions reflected a general improvement: 51.3% (99/193) showed moderate stress and 46.6% (90/193) reported low stress. The intracluster analysis revealed that under the intention-to-treat approach (Multimedia Appendix 6), only cluster 1 experienced a significant pre- versus postintervention effect of moderate size (Cohen *d*=0.46), while a larger pre- versus second postintervention assessment effect was observed in clusters 1 (Cohen *d*=0.68) and 5 (Cohen *d*=0.62). These effects were maintained in subsequent assessments. Under the per-protocol approach, the same effects were observed with larger effect sizes (Multimedia Appendix 7). Figure 2 shows the evolution of perceived stress along the 5 assessment points for each cluster (intention-to-treat approach).

**Table 2:** Effects of the intervention comparing intervention phase versus control phase (linear mixed-effects models) under the intention-to-treat and per-protocol approaches.

Outcome		Intention-to-treat approach		Per-protocol approach	
		Estimate (SE; 95% CI)	*P* value	Estimate (SE; 95% CI)	*P* value
PSS^a,b,c,d,e,^^f^		–1.08 (0.51; –2.08 to –0.08)	.03^g^	–1.84 (0.62; –3.04 to –0.63)	.003^g^
PHQ-9^a,c,d,e,^^h^		–1.43 (.030; –2.02 to –0.84)	<.001^g^	–1.72 (0.24; –2.19 to –1.26)	<.001^g^
GAD-7^a,c,d,e,i^		–1.31 (0.41; –2.12 to –0.51)	.001^g^	–1.65 (0.17; –1.98 to –1.31)	<.001^g^
**BSI-18^j^**					
	Somatization^a,c,d,e^	–0.73 (0.25; –1.22 to –0.24)	.004^g^	–0.83 (0.28; –1.38 to –0.29)	.003^g^
	Depression^a,d,e^	–1.06 (0.28; –1.62 to –0.51)	<.001^g^	–1.50 (0.36; –2.20 to –0.80)	<.001^g^
	Anxiety^a,c,d,e^	–0.76 (0.40; –1.55 to 0.02)	.06	–1.34 (0.16; –1.65 to –1.03)	<.001^g^
	GSI^a,d,e,k^	–2.30 (0.82; –3.92 to –0.69)	.005^g^	–3.60 (0.56; –4.71 to –2.50)	<.001^g^
	CD-RISC^a,c,d,e,^^l^	1.53 (0.47; 0.62 to 2.45)	.001^g^	2.14 (0.47; 1.22 to 3.06)	<.001^g^
**FFMQ^m^**					
	Observing^a,b,c,d,e^	0.16 (0.08; –0.01 to 0.32)	.07	0.36 (0.11; 0.14 to 0.57)	.001^g^
	Describing^a,c,d,n,o^	0.07 (0.14; –0.20 to 0.36)	.61	0.03 (0.09; –0.15 to 0.20)	.77
	Acting with awareness^a,d,e^	0.16 (0.09; –0.02 to 0.34)	.08	0.22 (0.05; 0.11 to 0.32)	<.001^g^
	Nonjudging^a,c,d,e^	0.27 (0.10; 0.08 to 0.47)	.007^g^	0.34 (0.09; 0.17 to 0.52)	<.001^g^
	Nonreacting^a,c^	0.18 (0.08; 0.03 to 0.33)	.02^g^	0.20 (0.07; 0.06 to 0.34)	.006^g^
**SOCS^p^**					
	Others^a,c,d,e^	–0.17 (0.70; –1.55 to 1.20)	.80	–0.07 (0.76; –1.56 to 1.42)	.93
	Self^a,c,e^	2.72 (0.66; 1.42 to 4.03)	<.001^g^	2.97 (0.82; 1.36 to 4.57)	<.001^g^
	AAQ-II^a,c,d,e,^^q^	–1.51 (0.74; –2.95 to –0.07)	.04	–1.54 (0.83; –3.17 to 0.09)	.06

^a^Baseline level of the outcome was significant in the model.

^b^Sequence was significant in the model.

^c^Type of contract was significant in the model.

^d^Occupation was significant in the model.

^e^Salary was significant in the model.

^f^PSS: Perceived Stress Scale.

^g^Effects that remained statistically significant (*P*<.05) after applying the Benjamini-Hochberg correction (applied in the secondary outcomes and process variables).

^h^PHQ-9: Patient Health Questionnaire-9.

^i^GAD-7: Generalized Anxiety Disorder-7.

^j^BSI-18: Brief Symptoms Inventory-18.

^k^GSI: Global Severity Index.

^l^CD-RISC: Connor-Davidson Resilience Scale.

^m^FFMQ: Five Facet Mindfulness Questionnaire-15.

^n^Age was significant in the model.

^o^Education level was significant in the model.

^p^SOCS: Sussex-Oxford Compassion Scales.

^q^AAQ-II: Acceptance and Action Questionnaire-II.

### Effects on Secondary Outcomes and Process Variables

As shown in Table 2, the effects of the intervention in all the secondary outcomes (PHQ-9, GAD-7, and BSI) were significant according to the linear mixed-effects models under the intention-to-treat approach, except for BSI-anxiety. Certain covariates played a significant role in the models: the baseline level of the outcome, type of contract, occupation, and salary. Under the per-protocol approach, all the secondary outcomes, including BSI-anxiety, presented a statistically significant effect, and the effects of the covariates were maintained in all cases.

Some process variables experienced significant improvement during the intervention phases: under the intention-to-treat approach, resilience (CD-RISC), the mindfulness facets of nonjudging and not reacting to the inner experience (FFMQ-15), and self-compassion (SOCS-self) presented significant effects. These were also observed under the per-protocol approach, along with significant effects on the mindfulness facets of observing and acting with awareness (FFMQ). All these effects were in the expected direction (improved during the intervention phase), and some covariates presented significant effects in each case (Table 2): baseline level of the outcome, the sequence in which the cluster was allocated, type of contract, occupation, salary, age, and education level.

## Discussion

### Principal Findings

The MINDxYOU program led to a significant reduction in health professionals’ perceived stress. Given the implications of sustained stress on these professionals’ well-being, this reduction can have a notable impact on preventing mental health issues, such as burnout syndrome and its associated health and societal consequences [9-13]. This finding is in line with those of previous studies conducted on smaller samples in which self-guided web-based programs implementing third-wave psychotherapy contents produced significant improvements in health professionals’ stress. Barrett and Stewart [63] reported that a web-based acceptance and commitment therapy program was as effective as a web-based CBT program, and the mindful and self-compassion program was superior when compared to a waitlist control group [24]. Similarly, 2 uncontrolled studies observed intragroup effects of similar interventions [64,65]. As far as the duration of the effects of the MINDxYOU program is concerned, these were maintained in those clusters where postintervention effects were found (clusters 1 and 5) across subsequent assessments, which suggests that the program’s effects might not be limited to the short term. Previous studies reported similar findings [64,66], but methodologically sound studies are required to ensure the long-term benefits of self-guided web-based programs in this population.

Likewise, the program produced significant improvements in mental health–related outcomes. Anxiety and depressive symptomatology were reduced after the intervention, which is in line with previous eHealth CBT-based interventions [67,68] and third-wave psychotherapies [69,70]. However, the effects of such interventions on somatic symptoms have not been as widely studied in this population, despite the numerous studies that reported increased somatization in health professionals during the COVID-19 pandemic [6]. The MINDxYOU program produced a significant reduction in somatic symptoms, which is in line with studies testing eHealth interventions on patients with somatic symptom disorder [71,72], but these findings should be extended in further studies conducted on health professionals. Considering the high frequency with which health professionals experience anxiety, depressive, and somatic symptoms, especially following the COVID-19 pandemic [3-6], the effectiveness of this intervention in reducing these symptoms represents a significant contribution to mental health promotion and supports its implementation in real-world settings.

It is hypothesized that improvements in perceived stress, depressive symptomatology, anxiety, and somatization might have been mediated by the changes produced in process variables, such as resilience, different mindfulness facets, and self-compassion. The MINDxYOU program aimed to promote mindfulness, that is, present-focused nonjudging attention, and self-compassion, that is, a kind attitude toward the self, both associated with reductions in anxiety and depression [73,74]. Third-wave psychotherapies have also been found to produce a significant increase in resilience [21], although not as clearly in the case of health professionals [75]. Interestingly, resilience mediates the association between mindfulness and self-compassion with depressive symptomatology [76], which highlights the inseparable nature of our study outcomes. We will be exploring the mediation paths of the MINDxYOU program in a future work to complement these findings. It is worth noting that other process variables, such as compassion for others and acceptance, did not experience significant improvements, despite the observation in previous studies that mindfulness-based interventions and eHealth programs might foster these outcomes [77,78]. Therefore, it could be hypothesized that our sample’s relatively high baseline degrees of acceptance and compassion for others produced a ceiling effect.

All in all, our findings seem to add evidence to the potential of self-guided, third-wave psychotherapy–based eHealth interventions to help health professionals with their stress and improve their mental health. This improvement can significantly impact job satisfaction and work performance, ultimately benefiting the entire population [33]. The self-guided format offers high levels of availability, flexibility, and convenience [26,27], and third-wave psychotherapies have widely proved their effectiveness on numerous health outcomes and in different populations, including health professionals [18,19,23]. Nevertheless, it should be noted that the effects of the program on every outcome were stronger when considering the per-protocol approach (the sample of intervention completers). Different studies have observed a significant association between an eHealth program’s dose and its effects on stress, among other outcomes [24,66,79,80], which highlights the importance of fostering treatment adherence.

In this regard, it is noteworthy that some differences were found between the individuals who initiated the program and those who did not; these reflect that some clusters were more active than others, suggesting different degrees of motivation toward the intervention. Moreover, program completion also showed some differences between clusters. Therefore, it is hypothesized that waiting time (the time between the baseline evaluation and accessing the program) may have been a barrier for some participants in our study, as also identified by previous works [81]; the stepped-wedge design meant that while some participants (clusters 1 and 5) accessed the intervention after a relatively short time (8 weeks), others had to wait up to 16 weeks (clusters 3 and 4) or 24 weeks (clusters 2 and 6). Furthermore, clusters 3 and 4 were given access to the intervention over the summer, which was probably not helpful for adherence as it coincided with the vacation period. In a future study, we will explore how these and other factors, such as common job stressors (eg, workload and work-family balance) [82], could have hindered adherence to the MINDxYOU program.

Hence, the study design might have been a hindrance to part of our sample initiating the program or continuing with it. Once the intervention had commenced, adherence to the program was moderate. Approximately 1 (50%) in every 2 participants who started the MINDxYOU program completed it, and they reported notable indicators of autonomous practice (around 3 days of formal practice per week, 19 minutes per practice, and a similar frequency for informal practice). To our knowledge, no previous studies testing the effectiveness of self-guided, third-wave psychotherapy–based web-based interventions have reported the amount of meditation practice; however, a recent systematic review concluded that patients with chronic pain who are undergoing in-person programs practiced an average of 4 days per week and 27 minutes per exercise [83]. These findings could suggest that web-based interventions might have a smaller yet sufficient impact on autonomous practice compared to third-wave psychotherapy programs with a conventional in-person format, but this remains a hypothesis to be explored in the future.

With regard to program use, MINDxYOU was accessed by two-thirds (229/357, 64.1%) of our sample with an average of 2.2 (SD 1.8) modules completed (out of 4), which meant logging into the web-based platform at least 4 times, because each module contained 2 sessions. This can be considered an acceptable rate of use and higher than that observed in the few studies that have previously reported on this aspect. Ketelaar et al [84] found that only 20% of their participants logged into their eHealth programs at least once, and none completed the intervention. Dutton and Kozachik [85] and Hersch et al [86], who tested the 8-week self-guided BREATHE web-based intervention on nurses, observed an average of 2 to 2.5 logins. Kemper et al [87] and Kemper and Khirallah [88] found that participants engaged with a median of 3 modules out of 12 (mind-body skills training).

The weekly contact with the research team may have been a reason for the notable use of the MINDxYOU program. This contact was included as part of the intervention based on the recommendations of previous studies [23,89] to prevent low adherence rates. However, emails and text messages, which were the most common form of contact option chosen, were often one-sided (ie, unanswered by the participant), which leaves room to doubt whether these contacts were useful in their function of enhancing adherence to the program. It could also be argued that the specific nature of the MINDxYOU program, which was designed particularly for health professionals, could be a reason for its good rates of use. To our knowledge, only 5 interventions with specific contents for health professionals have been tested [85,86,90-93], with heterogeneous effects, and only the BREATHE program reported its use, which was relatively low, as previously explained. Therefore, the benefits of implementing specific interventions for this population over generic ones remain a topic of debate and will require further studies.

### Strengths and Limitations

This study is part of the MINDxYOU project, which focuses on the well-being of health professionals, a topic that gained a notable degree of attention from researchers during the COVID-19 pandemic but is now at risk of again being underestimated and understudied now that the COVID-19 pandemic is over. This work reports part of the findings of the project that will need to be complemented with others (eg, mediation analysis, cost-effectiveness of the intervention, and implementation in health facilities). Altogether, these findings will contribute to the understanding of health professionals’ needs and address them using innovative, evidence-based interventions.

Another notable strength of this study is its sample size. An effort was made to recruit almost twice the sample that was required according to the sample size calculation [33], which turned out to be very valuable considering the high rate of attrition during the study. While previous randomized controlled trials have tested the effectiveness of eHealth programs on health professionals, only 1 study was conducted on a larger sample [93]; however, no significant effects of the intervention—a mobile app (PsyCovid)—were found compared to the control group, which is in line with other studies testing mobile apps [23]. Our study analyzed the effects of a self-guided web-based intervention, a format that has shown good results in previous studies [24,63-65], and was developed based on previously validated interventions but with a special focus on health workers’ stressors, which was suggested to be an important aspect for increasing adherence to the program [23,29]. In addition, our study provides data on adherence to the intervention in terms of frequency and duration of autonomous practice, which is a valuable indicator of the degree to which individuals (in this case, health professionals) can implement what they learn in self-guided web-based interventions.

Nevertheless, this study does have some notable limitations. First, the study may have been affected by self-selection bias, as only individuals who expressed interest in the project, potentially due to experiencing certain levels of stress or having a particular interest in the meditation techniques offered by the intervention, were eligible to participate. In addition, some of the criteria that were used to include or exclude potential participants may have affected the representativeness of our sample. On one hand, individuals diagnosed with a severe mental health condition (depression with active suicidal ideation, bipolar disorder, etc) were excluded and redirected to a specialized service. By contrast, having prospects of staying at the same workplace for the following 6 months was an inclusion criterion because it was considered relevant to studying the program implementation, but this led to health professionals with unstable employment situations being excluded. Job instability is clearly associated with stress and, added to the exclusion of individuals with severe mental health conditions, this could partially explain the relatively low levels of psychopathological symptoms (anxiety, depression, and somatization) in our sample, which probably had an overrepresentation of healthy individuals with stable employment. Similarly, the study was restricted to health professionals working in 2 Spanish regions, and generalizations in this regard could also be limited as each autonomous region in Spain manages its own health and social care provision. Therefore, regional differences may exist in terms of health resources or expenditure, which could undoubtedly affect the day-to-day activities of health professionals in each region and cater to their particular needs.

It should also be noted that our stepped-wedge cluster randomized design presented some limitations. The first one is that the clusters were not balanced in terms of sample size, which was due to the type of workplace. While hospitals employ a large number of health professionals, primary care centers and nursing homes tend to have smaller staffing needs. Moreover, because of this design, even though every study participant was assessed both before and after the intervention, not all of them (ie, clusters 2 and 6) were assessed again after the first postintervention evaluation. This hinders the extraction of conclusions regarding the medium- or long-term effects of the intervention, which is a very important aspect to be considered in future studies. The different waiting times for each cluster can also be considered a limitation because it could have negatively affected the motivation of some study participants toward the intervention, hindering adherence. In this regard, it is important to note that a notable portion of the study participants (128/357, 35.9%) did not initiate this self-guided program, which may reflect the presence of barriers (eg, multitasking, heavy workloads, and constant evaluations) that will be explored in future research. Another limitation of this study is the use of self-reported measures, as these imply a certain bias, although most of them presented strong internal validity at the different assessment points. Complementing the assessment with some physiological outcomes could be considered in future studies.

### Conclusions

Our findings support the effectiveness of the MINDxYOU program for reducing stress and promoting mental health (ie, reducing depressive, anxiety, and somatic symptoms and increasing resilience, mindfulness facets, and self-compassion) in health professionals. The intervention dose seems to play a significant role in its effects, which highlights the need for increased adherence to the program. Although we hypothesize that the study design might have hindered adherence in some cases, program use was notable compared to that reported by previous studies testing similar eHealth programs, which could be partially due to the inclusion of a small degree of guidance (ie, weekly contact from the research team during the intervention). These findings will need to be complemented with the study of the cost-effectiveness of the MINDxYOU program and its implementation in different health facilities to consider its potential as an efficient and reliable tool for delivery in Spanish health care institutions.

## Appendix

Multimedia Appendix 1 Outline of the contents of the MINDxYOU program.

Multimedia Appendix 2 Baseline differences between participants who completed all the assessments and those who did not.

Multimedia Appendix 3 Baseline differences between participants who started the web-based program and those who did not.

Multimedia Appendix 4 Baseline differences between participants who completed the web-based program (ie, at least 3 of the 4 modules) and those who did not.

Multimedia Appendix 5 Baseline characteristics of the study sample.

Multimedia Appendix 6 Intracluster effects in the main outcome (Perceived Stress Scale). Intention-to-treat approach.

Multimedia Appendix 7 Intracluster effects in the main outcome (Perceived Stress Scale). Per-protocol approach.

Multimedia Appendix 8 CONSORT (Consolidated Standards of Reporting Trials) eHEALTH checklist (V 1.6.1).

## Abbreviations

AAQ-II: Acceptance and Action Questionnaire-II
BSI-18: Brief Symptoms Inventory-18
CBT: cognitive behavioral therapy
CD-RISC: Connor-Davidson Resilience Scale
CONSORT: Consolidated Standards of Reporting Trials
FFMQ-15: Five Facet Mindfulness Questionnaire-15
GAD-7: Generalized Anxiety Disorder-7
PHQ-9: Patient Health Questionnaire-9
PSS: Perceived Stress Scale
SOCS: Sussex-Oxford Compassion Scales

## Data Availability

The datasets generated and analyzed during this study are available from the corresponding author on reasonable request.
