# Supplementary material for: Effectiveness of a Web-Based Self-Guided Intervention (MINDxYOU) for Reducing Stress and Promoting Mental Health Among Health Professionals: Results From a Stepped-Wedge Cluster Randomized Trial
Source: J Med Internet Res. 2025 Feb 3;27:e59653. doi: 10.2196/59653 (PMC11833273; doi:10.2196/59653)
Supplement: Multimedia Appendix 1 [file jmir_v27i1e59653_app1.docx]

**Supplementary** **table 1.** Outline of the contents of the MINDxYOU program.

| Module | Session | Contents | Formal practice | Informal practice |
| --- | --- | --- | --- | --- |
| Module 0 | - | • 3-minute video-tutorial (how to navigate the online platform, recommendations for using the program)  • What is MINDxYOU?  • Who is MINDxYOU aimed at?  • How to use MINDxYOU?  • Motivation and expectations | - | - |
| Module 1 | Session 1: Getting to know mindfulness | • Multitask mode  • The internal dialogue  • What is mindfulness?  • How does it work?  • How to start practicing it? | • Mindful breathing^1^  • Three-step practice^1^ | - |
|  | Session 2: Training our mind | • Neurological effects of meditation  • Fusion with thoughts  • Voluntary and involuntary thoughts  • How to detach from our thoughts? | • Body scan^2^ | - |
| Module 2 | Session 1: Getting to know compassion | • Compassion and self-compassion in psychology  • Therefore, what is NOT compassion?  • Mindfulness and compassion  • How to start developing compassion | • Compassionate breathing^3^  • Compassionate three-step practice^3^ | - |
|  | Session 2: Developing a compassionate approach | • Compassion fatigue in health professionals  • Primary and secondary suffering  • Biological bases of compassion and stress  • Psychological impact of compassion  • Benefits of practicing compassion | • Safe place meditation^1^  • Compassionate gestures + compassionate words^1^ | - |
| Module 3 | Session 1: Getting to know acceptance | • What is acceptance?  • Basic principles of acceptance  • Obstacles for acceptance  • The role of acceptance in health professionals | • Forgiving the pain meditation^4^ | - |
|  | Session 2: Personal values and committed actions | • Personal values  • Committed actions  • Acceptance in personal relationships and interactions with patients  • Not taking things personally | • Old man/woman meditation^4^ | - |
| Module 4 | Session 1: Applying mindfulness and compassion to physical activity and rest | • Physical activity: introduction  • Common obstacles to remaining active  • How to apply mindfulness in this habit?  • Resting: introduction  • Common obstacles to resting  • How to apply mindfulness to this habit? | - | • Applying mindfulness when doing physical activity (e.g., walking, sports, etc.)^5^  • Applying mindfulness to both falling asleep and resting when tired.^5^ |
|  | Session 2: Applying mindfulness and compassion to have a healthy diet and social life | • Healthy diet: introduction  • Common obstacles to maintaining a healthy diet  • How to apply mindfulness in this habit?  • Socializing: introduction  • Common obstacles to being social  • How to apply mindfulness in this habit? | - | • Applying mindfulness when eating (i.e., mindful eating).^5^  • Applying mindfulness and compassion when interacting with others.^5^ |

***Note***: Recommendations for frequency of practice were ^1^ once per day during the following 7 days, ^2^ at least 4 times in the following 7 days, ^3^ once per day during the following 7 days in substitution of the exercises that were practiced previously (i.e., mindful breathing and the three-step practice), ^4^ once during the following 7 days, and ^5^ every time that the participant had the chance
