# Supplementary material for: Effectiveness of a Web-Based Self-Guided Intervention (MINDxYOU) for Reducing Stress and Promoting Mental Health Among Health Professionals: Results From a Stepped-Wedge Cluster Randomized Trial
Source: J Med Internet Res. 2025 Feb 3;27:e59653. doi: 10.2196/59653 (PMC11833273; doi:10.2196/59653)
Supplement: Multimedia Appendix 2 [file jmir_v27i1e59653_app2.docx]

**Supplementary table 2**. Baseline differences between participants who completed all the assessments and those who did not.

|  | Completed all assessments  (n = 131) | Did not complete all assessments  (n = 226) | *t* or χ^2^ (*P*) |
| --- | --- | --- | --- |
| Sociodemographic characteristics | | | |
| Cluster, n (%)  · Cluster 1 (Aragón’s hospitals)  · Cluster 2 (Aragón’s PC centers)  · Cluster 3 (Aragón’s other centers)  · Cluster 4 (Málaga’s hospitals)  · Cluster 5 (Málaga PC centers)  · Cluster 6 (Málaga’s other centers) | 41 (48.8%)  24 (49%)  20 (33.3%)  14 (17.9%)  25 (41.7%)  7 (26.9%) | 43 (51.2%)  25 (51%)  40 (66.7%)  64 (82.1%)  35 (58.3%)  19 (73.1%) | **22.29 (.001)** |
| Sex, n (%)  · Females  · Males | 114 (38.4%)  17 (33.33%) | 183 (61.6%)  34 (66.7%) | 0.47 (.49) |
| Age, M (SD) | 47.10 (10.77) | 43.75 (11.25) | -2.73 (.007) |
| Region, n (%)  · Aragón  · Málaga | 85 (44%)  46 (28%) | 108 (56%)  118 (72%) | **9.76 (.002)** |
| Marital status, n (%)  · Married  · Single  · Divorced  · Widowed | 101 (38.8%)  20 (33.3%)  10 (41.7%)  0 (0%) | 159 (61.2%)  40 (66.7%)  14 (58.3%)  3 (100%) | 2.61 (.46) |
| Education level, n (%)  · Primary  · Secondary  · University | 4 (22.2%)  9 (36%)  18 (38.8%) | 14 (77.8%)  16 (64%)  186 (61.2%) | 2.22 (.53) |
| Work-related aspects | | | |
| Workplace, n (%)  · Hospital  · Primary care center  · Others | 61 (37.2%)  40 (41.2%)  30 (32.3%) | 103 (62.8%)  57 (58.8%)  63 (67.7%) | 1.65 (.44) |
| Type of contract, n (%)  · Functionary  · Indefinite  · Temporary (< 6 months)  · Temporary (> 6 months)  · Others | 69 (43.4%)  16 (29.1%)  3 (14.3%)  12 (27.9%)  31 (44.9%) | 90 (56.6%)  39 (70.9%)  18 (85.7%)  31 (72.1%)  38 (55.1%) | 14.27 (.03) |
| Occupation, n (%)  · Physician  · Nurse  · Nursing assistant  · Physiotherapist  · Psychologist  · Others | 58 (39.5%)  40 (43%)  11 (31.4%)  7 (36.8%)  5 (29.4%)  10 (27.8%) | 89 (60.5%)  53 (57%)  24 (68.6%)  12 (63.2%)  12 (70.6%)  26 (72.2%) | 4.24 (.64) |
| Management position, n (%)  · No  · Yes | 114 (38.6%)  17 (32.7%) | 181 (61.4%)  35 (67.3%) | 0.66 (.41) |
| Trainee, n (%)  · No  · Yes | 123 (38.1%)  8 (23.5%) | 200 (61.9%)  26 (76.5%) | 2.80 (.09) |
| Salary, n (%)  · Less than the minimum wage  · 1-2 times the minimum wage  · 2-3 times the minimum wage  · > 3 times the minimum wage | 1 (33.3%)  47 (32.2%)  52 (42.6%)  31 (40.8%) | 2 (66.7%)  99 (67.8%)  70 (57.4%)  45 (59.2%) | 3.48 (.32) |
| Clinical variables, M (SD) [score range] | | | |
| PSS [0 – 40] | 17.18 (6.58) | 16.71 (6.18) | -0.67 (.51) |
| PHQ-9 [0 – 27] | 6.75 (4.59) | 5.95 (4.20) | -1.66 (.10) |
| GAD-7 [0 – 21] | 7.77 (4.28) | 6.61 (4.14) | -2.51 (.01) |
| BSI-18  · Somatization [0 – 24]  · Depression [0 – 24]  · Anxiety [0 – 24]  · Total [0 – 72] | 3.40 (3.71)  4.85 (4.65)  5.18 (4.01)  13.42 (10.67) | 2.83 (3.36)  4.30 (3.86)  4.56 (3.72)  11.69 (9.33) | -1.47 (.14)  -1.14 (.26)  -1.45 (.15)  -1.59 (.11) |
| Process variables, M (SD) [score range] | | | |
| CD-RISC [0 – 40] | 26.39 (7.14) | 27.87 (6.53) | 1.97 (.049) |
| FFMQ-15 [1 – 5]  · Observing  · Describing  · Acting with awareness  · Nonjudging  · Nonreacting | 2.92 (0.82)  3.48 (0.85)  3.15 (0.95)  3.73 (0.92)  2.99 (0.77) | 2.80 (0.88)  3.58 (0.83)  3.33 (0.87)  3.84 (0.84)  3.02 (0.89) | -1.33 (.18)  1.06 (.29)  1.81 (.07)  1.06 (.29)  0.40 (.69) |
| SOCS [20 – 100]  · Compassion for others  · Self-compassion | 61.95 (8.50)  52.32 (10.49) | 61.86 (8.96)  54.27 (10.04) | -0.10 (.92)  1.72 (.09) |
| AAQ-II [7 – 49] | 21.24 (8.66) | 20.68 (8.14) | -0.61 (.52) |

***Note***: in **bold**, effects that remained statistically significant (i.e., *P* < .05) after applying the Benjamini-Hochberg correction for multiple tests.
