# Supplementary material for: Effectiveness of a Web-Based Self-Guided Intervention (MINDxYOU) for Reducing Stress and Promoting Mental Health Among Health Professionals: Results From a Stepped-Wedge Cluster Randomized Trial
Source: J Med Internet Res. 2025 Feb 3;27:e59653. doi: 10.2196/59653 (PMC11833273; doi:10.2196/59653)
Supplement: Multimedia Appendix 3 [file jmir_v27i1e59653_app3.docx]

**Supplementary table 3**. Baseline differences between participants who started the web-based program and those who did not.

|  | Started the program  (n = 229) | Did not start the program  (n = 128) | *t* or χ^2^ (*P*) |
| --- | --- | --- | --- |
| Sociodemographic characteristics | | | |
| Cluster, n (%)  · Cluster 1 (Aragón’s hospitals)  · Cluster 2 (Aragón’s PC centers)  · Cluster 3 (Aragón’s other centers)  · Cluster 4 (Málaga’s hospitals)  · Cluster 5 (Málaga PC centers)  · Cluster 6 (Málaga’s other centers) | 65 (77.4%)  31 (63.3%)  42 (70%)  36 (46.2%)  41 (68.3%)  14 (53.4%) | 19 (22.6%)  18 (36.7%)  18 (30%)  42 (53.8%)  19 (31.7%)  12 (46.2%) | **19.94 (.001)** |
| Sex, n (%)  · Females  · Males | 190 (64%)  32 (62.7%) | 107 (36%)  19 (37.3%) | 0.03 (.87) |
| Age, M (SD) | 45.80 (11.41) | 43.62 (10.65) | -1.74 (.08) |
| Region, n (%)  · Aragón  · Málaga | 138 (71.5%)  91 (55.5%) | 55 (28.5%)  73 (44.5%) | **9.89 (.002)** |
| Marital status, n (%)  · Married  · Single  · Divorced  · Widowed | 175 (67.3%)  31 (51.7%)  14 (58.3%)  2 (66.7%) | 85 (32.7%)  29 (48.3%)  10 (41.7%)  1 (33.3%) | 5.54 (.14) |
| Education level, n (%)  · Primary  · Secondary  · University | 11 (61.1%)  15 (60%)  196 (64.5%) | 7 (38.9%)  10 (40%)  108 (35.5%) | 1.51 (.68) |
| Work-related aspects | | | |
| Workplace, n (%)  · Hospital  · Primary care center  · Others | 104 (63.4%)  63 (64.9%)  60 (64.5%) | 60 (36.6%)  34 (35.1%)  33 (35.5%) | 0.07 (.97) |
| Type of contract, n (%)  · Functionary  · Indefinite  · Temporary (< 6 months)  · Temporary (> 6 months)  · Others | 105 (66%)  35 (63.6%)  11 (52.4%)  25 (58.1%)  2 (100%) | 54 (34%)  20 (36.4%)  10 (47.6%)  18 (41.9%)  0 (0%) | 3.37 (.76) |
| Occupation, n (%)  · Physician  · Nurse  · Nursing assistant  · Physiotherapist  · Psychologist  · Others | 103 (70.1%)  55 (59.1%)  23 (65.7%)  11 (57.9%)  12 (70.6%)  18 (50%) | 44 (29.9%)  38 (40.9%)  12 (34.3%)  8 (42.1%)  5 (29.4%)  18 (50%) | 8.15 (.23) |
| Management position, n (%)  · No  · Yes | 194 (65.8%)  28 (53.8%) | 101 (34.2%)  24 (46.2%) | 2.72 (.10) |
| Trainee, n (%)  · No  · Yes | 206 (63.8%)  23 (67.6%) | 117 (36.2%)  11 (32.4%) | 0.20 (.65) |
| Salary, n (%)  · Less than the minimum wage  · 1-2 times the minimum wage  · 2-3 times the minimum wage  · > 3 times the minimum wage | 2 (66.7%)  87 (59.6%)  86 (70.5%)  47 (61.8%) | 1 (33.3%)  59 (40.4%)  36 (29.5%)  29 (38.2%) | 3.63 (.31) |
| Clinical variables, M (SD) [score range] | | | |
| PSS [0 – 40] | 16.76 (6.46) | 17.10 (6.12) | 0.48 (.63) |
| PHQ-9 [0 – 27] | 6.40 (4.34) | 5.98 (4.41) | -0.85 (.39) |
| GAD-7 [0 – 21] | 7.33 (4.24) | 6.54 (4.17) | -1.69 (.09) |
| BSI-18  · Somatization [0 – 24]  · Depression [0 – 24]  · Anxiety [0 – 24]  · Total [0 – 72] | 3.01 (3.36)  4.65 (4.44)  5.03 (3.77)  12.69 (9.88) | 3.10 (3.75)  4.24 (3.68)  4.38 (3.93)  11.71 (9.90) | 0.21 (.83)  -0.93 (.35)  -1.52 (.13)  -0.89 (.38) |
| Process variables, M (SD) [score range] | | | |
| CD-RISC [0 – 40] | 27.12 (7.05) | 27.65 (6.32) | 0.69 (.49) |
| FFMQ-15 [1 – 5]  · Observing  · Describing  · Acting with awareness  · Nonjudging  · Nonreacting | 2.89 (0.81)  3.54 (0.82)  3.20 (0.92)  3.74 (0.89)  3.09 (0.79) | 2.76 (0.93)  3.55 (0.87)  3.37 (0.88)  3.90 (0.83)  2.88 (0.93) | -1.29 (.20)  0.11 (.91)  1.60 (.11)  1.63 (.11)  -2.22 (.03) |
| SOCS [20 – 100]  · Compassion for others  · Self-compassion | 62.17 (8.38)  53.06 (10.05) | 61.41 (9.45)  54.38 (10.57) | -0.77 (.44)  1.15 (.25) |
| AAQ-II [7 – 49] | 21.55 (8.46) | 19.72 (7.99) | -1.98 (.049) |

***Note***: in **bold**, effects that remained statistically significant (i.e., *P* < .05) after applying the Benjamini-Hochberg correction for multiple tests.
