# Supplementary material for: Effectiveness of a Web-Based Self-Guided Intervention (MINDxYOU) for Reducing Stress and Promoting Mental Health Among Health Professionals: Results From a Stepped-Wedge Cluster Randomized Trial
Source: J Med Internet Res. 2025 Feb 3;27:e59653. doi: 10.2196/59653 (PMC11833273; doi:10.2196/59653)
Supplement: Multimedia Appendix 4 [file jmir_v27i1e59653_app4.docx]

**Supplementary table 4**. Baseline differences between participants who completed the web-based program (i.e., at least 3 of the 4 modules) and those who did not.

|  | Completed the program  (n = 112) | Did not complete the program  (n = 119) | *t* or χ^2^ (*P*) |
| --- | --- | --- | --- |
| Sociodemographic characteristics | | | |
| Cluster, n (%)  · Cluster 1 (Aragón’s hospitals)  · Cluster 2 (Aragón’s PC centers)  · Cluster 3 (Aragón’s other centers)  · Cluster 4 (Málaga’s hospitals)  · Cluster 5 (Málaga PC centers)  · Cluster 6 (Málaga’s other centers) | 35 (53.8%)  16 (51.6%)  20 (47.6%)  13 (36.1%)  24 (58.5%)  4 (28.6%) | 30 (46.2%)  15 (48.4%)  22 (52.4%)  23 (63.9%)  17 (41.5%)  10 (71.4%) | 6.95 (.22) |
| Sex, n (%)  · Females  · Males | 95 (50%)  15 (46.9%) | 95 (95%)  17 (53.1%) | 0.11 (.74) |
| Age, M (SD) | 47.98 (10.30) | 43.65(12.07) | -2.87 (.004) |
| Region, n (%)  · Aragón  · Málaga | 71 (51.4%)  41 (45.1%) | 67 (48.6%)  50 (54.9%) | 0.90 (.34) |
| Marital status, n (%)  · Married  · Single  · Divorced  · Widowed | 87 (49.7%)  13 (41.9%)  10 (71.4%)  0 (0%) | 88 (50.3%)  18 (16.1%)  4 (28.6%)  2 (100%) | 5.37 (.15) |
| Education level, n (%)  · Primary  · Secondary  · University | 6 (54.5%)  8 (53.3%)  97 (49.5%) | 5 (45.5%)  7 (46.7%)  99 (50.5%) | 0.76 (.86) |
| Work-related aspects | | | |
| Workplace, n (%)  · Hospital  · Primary care center  · Others | 50 (48.1%)  34 (54%)  28 (46.7%) | 54 (51.9%)  29 (46%)  32 (53.3%) | 0.78 (.68) |
| Type of contract, n (%)  · Functionary  · Indefinite  · Temporary (< 6 months)  · Temporary (> 6 months)  · Others | 57 (54.3%)  12 (34.3%)  4 (36.4%)  12 (48%)  21 (45.7%) | 48 (45.7%)  23 (65.7%)  7 (63.3%)  13 (52%)  25 (54.3%) | 7.43 (.28) |
| Occupation, n (%)  · Physician  · Nurse  · Nursing assistant  · Physiotherapist  · Psychologist  · Others | 46 (44.7%)  30 (54.5%)  11 (47.8%)  7 (63.6%)  6 (50%)  10 (55.6%) | 57 (55.3%)  25 (45.5%)  12 (52.2%)  4 (36.4%)  6 (50%)  8 (44.4%) | 2.70 (.75) |
| Management position, n (%)  · No  · Yes | 97 (50%)  13 (46.4%) | 97 (50%)  15 (53.6%) | 0.13 (.72) |
| Trainee, n (%)  · No  · Yes | 107 (51.9%)  5 (21.7%) | 99 (48.1%)  18 (78.3%) | 7.55 (.006) |
| Salary, n (%)  · Less than the minimum wage  · 1-2 times the minimum wage  · 2-3 times the minimum wage  · > 3 times the minimum wage | 1 (50%)  41 (47.1%)  44 (51.2%)  24 (51.1%) | 1 (50%)  46 (52.9%)  42 (48.8%)  23 (48.9%) | 0.34 (.95) |
| Clinical variables, M (SD) [score range] | | | |
| PSS [0 – 40] | 17.21 (6.70) | 16.32 (6.21) | -1.02 (.31) |
| PHQ-9 [0 – 27] | 6.78 (4.56) | 6.03 (4.10) | -1.30 (.20) |
| GAD-7 [0 – 21] | 7.74 (4.07) | 6.94 (4.38) | -1.41 (.16) |
| BSI-18  · Somatization [0 – 24]  · Depression [0 – 24]  · Anxiety [0 – 24]  · Total [0 – 72] | 3.41 (3.75)  5.21 (4.93)  5.22 (3.91)  13.84 (10.61) | 2.63 (2.89)  4.11 (3.84)  4.84 (3.63)  11.57 (9.01) | -1.75 (.08)  -1.86 (.06)  -0.75 (.46)  -1.72 (.09) |
| Process variables, M (SD) [score range] | | | |
| CD-RISC [0 – 40] | 26.28 (7.35) | 27.95 (6.68) | 1.77 (.08) |
| FFMQ-15 [1 – 5]  · Observing  · Describing  · Acting with awareness  · Nonjudging  · Nonreacting | 2.86 (0.83)  3.40 (0.77)  3.10 (0.94)  3.69 (0.91)  2.98 (0.78) | 2.92 (0.79)  3.67 (0.85)  3.30 (0.89)  3.79 (0.87)  3.19 (0.78) | 0.52 (.60)  2.40 (.02)  1.55 (.12)  0.92 (.36)  1.96 (.05) |
| SOCS [20 – 100]  · Compassion for others  · Self-compassion | 61.82 (8.03)  51.75 (9.82) | 62.51 (8.73)  54.35 (10.15) | 0.61 (.54)  1.9 (.05) |
| AAQ-II [7 – 49] | 22.24 (8.29) | 20.88 (8.61) | -1.19 (.24) |

***Note***: None of the effects remained statistically significant (i.e., *P* < .05) after applying the Benjamini-Hochberg correction for multiple tests.
