# Supplementary material for: Effectiveness of a Web-Based Self-Guided Intervention (MINDxYOU) for Reducing Stress and Promoting Mental Health Among Health Professionals: Results From a Stepped-Wedge Cluster Randomized Trial
Source: J Med Internet Res. 2025 Feb 3;27:e59653. doi: 10.2196/59653 (PMC11833273; doi:10.2196/59653)
Supplement: Multimedia Appendix 5 [file jmir_v27i1e59653_app5.docx]

**Supplementary table 5**. Baseline characteristics of the study sample.

|  | Cluster 1  (n = 81) | Cluster 2  (n = 48) | Cluster 3  (n = 60) | Cluster 4  (n = 75) | Cluster 5  (n = 57) | Cluster 6  (n = 26) | Total sample  (n = 347) | *F* or χ^2^ (*P*) |
| --- | --- | --- | --- | --- | --- | --- | --- | --- |
| Sociodemographic characteristics | | | | | | | | |
| Sex, n of females (%) | 73 (90.1%) | 44 (91.7%) | 55 (91.7%) | 59 (78.7%) | 44 (75.9%) | 22 (84.6%) | 297 (85.6%) | 11.79 (.04) |
| Age, M (SD) | 44.95 (10.42) | 47.60 (12.24) | 44.27 (10.23) | 42.81 (10.03) | 49.81 (11.32) | 38.00 (11.62) | 45.01 (11.17) | **5.66 (<.001)** |
| Marital status, n of married (%) | 62 (77.8%) | 36 (75%) | 49 (81.7%) | 54 (72%) | 43 (75.4%) | 15 (57.7%) | 260 (74.9%) | 19.11 (.21) |
| Education level, n (%)  · Primary/elementary  · Secondary  · University | 1 (1.2%)  8 (9.9%)  72 (88.9%) | -  4 (8.3%)  44 (91.7%) | 13 (21.7%)  6 (10%)  41 (68.3%) | 3 (4%)  1 (1.3%)  1 (94.7%) | 1 (1.8%)  3 (5.3%)  53 (93%) | -  3 (11.5%)  23 (88.5%) | 18 (5.2%)  25 (7.2%)  304 (87.6%) | **68.82 (<.001)** |
| Work-related aspects | | | | | | | | |
| Type of contract, n (%)  · Public sector (permanent)  · Indefinite  · Temporary (< 6 months)  · Temporary (> 6 months)  · Others | 40 (49.4%)  11 (13.6%)  3 (3.7%)  8 (9.9%)  19 (23.5%) | 25 (52.1%)  3 (6.3%)  2 (4.2%)  6 (12.5%)  12 (25%) | 28 (46.7%)  15 (25%)  -  7 (11.7%)  10 (16.7%) | 29 (38.7%)  7 (9.3%)  12 (16%)  13 (17.3%)  14 (4%) | 35 (61.4%)  3 (5.3%)  3 (5.3%)  6 (10.5%)  10 (17.5%) | 2 (7.7%)  16 (61.5%)  1 (3.8%)  3 (11.5%)  4 (15.3%) | 159 (45.8%)  55 (15.9%)  21 (6.1%)  43 (12.4%)  69 (19.9%) | **97.31 (<.001)** |
| Occupation, n (%)  · Physician  · Nurse  · Nursing assistant  · Physiotherapist  · Psychologist  · Others | 37 (45.7%)  18 (22.2%)  7 (8.6%)  11 (13.6%)  2 (2.5%)  6 (7.4%) | 32 (66.7%)  14 (29.2%)  -  -  1 (2.1%)  1 (2.1%) | 14 (23.3%)  11 (18.3%)  20 (33.3%)  3 (5%)  4 (6.7%)  8 (12.4%) | 36 (48%)  26 (34.7%)  4 (5.3%)  1 (1.3%)  2 (2.7%)  6 (8%) | 26 (45.6%)  19 (33.3%)  1 (1.8%)  1 (1.8%)  1 (1.8%)  9 (15.8%) | 2 (7.7%)  5 (19.2%)  3 (11.5%)  3 (11.5%)  7 (26.9%)  6 (23.1%) | 147 (42.4%)  93 (26.8%)  35 (10.1%)  19 (5.5%)  17 (4.9%)  36 (10.4%) | **131.23 (<.001)** |
| Management position, n (%) | 11 (13.6%) | 2 (4.2%) | 11 (18.3%) | 14 (18.7%) | 9 (15.8%) | 5 (19.2%) | 52 (15%) | 6.26 (.28) |
| Trainee, n (%) | 9 (10.7%) | 9 (18.4%) | - | 11 (14.1%) | 5 (8.3%) | - | 34 (9.8%) | **15.64 (.008)** |
| Salary, n (%)  · Less than the minimum wage  · 1-2 times the minimum wage  · 2-3 times the minimum wage  · > 3 times the minimum wage | -  31 (38.3%)  27 (33.3%)  23 (28.4%) | -  9 (18.8%)  22 (45.8%)  17 (35.4%) | 1 (1.7%)  41 (68.3%)  12 (20%)  6 (10%) | -  28 (37.3%)  26 (34.7%)  21 (28%) | 1 (1.8%)  17 (29.8%)  31 (54.4%)  8 (14%) | 1 (3.8%)  20 (76.9%)  4 (15.4%)  1 (3.8%) | 3 (0.9%)  146 (42.1%)  122 (35.2%)  76 (21.9%) | **62.30**  **(<.001)** |
| Clinical variables, M (SD) [score range] | | | | | | | | |
| PSS [0 – 40] | 17.98 (6.35) | 18.33 (6.02) | 16.42 (6.13) | 15.29 (5.64) | 16.53 (7.09) | 17.27 (6.75) | 16.88 (6.33) | 2.09 (.07) |
| PHQ-9 [0 – 27] | 6.81 (4.01) | 6.65 (4.46) | 6.00 (4.42) | 5.40 (4.24) | 6.35 (4.93) | 6.58 (4.17) | 6.25 (4.36) | 0.99 (.42) |
| GAD-7 [0 – 21] | 7.72 (3.79) | 7.46 (3.74) | 6.88 (4.41) | 6.11 (4.05) | 6.89 (4.96) | 7.62 (4.51) | 7.05 (4.23) | 1.37 (.23) |
| BSI-18  · Somatization [0 – 24]  · Depression [0 – 24]  · Anxiety [0 – 24]  · GSI [0 – 72] | 2.99 (3.43)  5.15 (4.30)  5.31 (3.84)  13.44 (9.74) | 3.40 (3.44)  5.23 (4.53)  5.58 (3.66)  14.21 (9.74) | 2.82 (3.17)  3.68 (3.63)  4.38 (3.41)  10.88 (8.80) | 2.60 (3.30)  3.60 (3.57)  4.16 (3.81)  10.36 (9.25) | 3.40 (3. 34)  4.60 (4.49)  4.60 (4.03)  12.60 (10.30) | 3.58 (5.20)  5.46 (4.74)  4.92 (4.57)  13.96 (12.86) | 3.04 (3.50)  4.50 (4.18)  4.79 (3.84)  12.34 (9.88) | 0.63 (.68)  2.16 (.06)  1.29 (.27)  1.57 (.17) |
| Process variables, M (SD) [score range] | | | | | | | | |
| CD-RISC [0 – 40] | 26.36 (7.01) | 25.65 (6.58) | 27.37 (7.13) | 28.83 (6.35) | 27.98 (6.55) | 27.38 (6.98) | 27.31 (6.79) | 1.77 (.12) |
| FFMQ-15 [1-5]  · Observing  · Describing  · Acting with awareness  · Nonjudging  · Nonreacting | 2.70 (0.74)  3.55 (0.79)  3.02 (0.86)  3.56 (0.88)  2.92 (0.78) | 2.73 (0.87)  3.33 (0.96)  3.13 (0.97)  3.78 (0.82)  2.91 (0.67) | 2.73 (0.85)  3.52 (0.96)  3.39 (0.92)  3.84 (0.87)  3.06 (0.85) | 2.98 (0.89)  3.65 (0.75)  3.47 (0.84)  4.00 (0.90)  3.05 (0.92) | 3.12 (0.87)  3.55 (0.82)  3.35 (0.84)  3.92 (0.83)  3.03 (0.95) | 2.81 (0.98)  3.59 (0.71)  3.17 (1.05)  3.62 (0.83)  3.23 (0.91) | 2.84 (0.86)  3.54 (0.84)  3.26 (0.91)  3.80 (0.87)  3.01 (0.85) | 2.34 (.04)  0.88 (.50)  2.55 (.03)  2.50 (.03)  0.73 (.60) |
| SOCS [20-100]  · Compassion for others  · Self-compassion | 61.56 (9.06)  50.96 (9.27) | 58.67 (9.41)  51.70 (10.79) | 62.80 (2.67)  53.30 (10.90) | 64.00 (7.44)  56.95 (9.70) | 61.82 (8.24)  53.88 (10.01) | 60.92 (8.94)  55.00 (10.42) | 61.90 (8.77)  53.53 (10.24) | 2.43 (.04)  **3.20 (.008)** |
| AAQ-II [7 – 49] | 22.77 (7.61) | 21.79 (5.59) | 20.75 (8.35) | 18.91 (6.69) | 20.60 (8.62) | 20.12 (7.52) | 20.89 (8.33) | 1.87 (.10) |

***Note***: in **bold**, effects that remained statistically significant (i.e., *P* < .05) after applying the Benjamini-Hochberg correction for multiple tests. Missing values were found in the next variables: CD-RISC (one in cluster 5), FFMQ (one in cluster 4, two in cluster 5), SOCS-Others (one in cluster 5), SOCS-Self (one in cluster 4, one in cluster 5)
