# Supplementary material for: Effectiveness of a Web-Based Self-Guided Intervention (MINDxYOU) for Reducing Stress and Promoting Mental Health Among Health Professionals: Results From a Stepped-Wedge Cluster Randomized Trial
Source: J Med Internet Res. 2025 Feb 3;27:e59653. doi: 10.2196/59653 (PMC11833273; doi:10.2196/59653)
Supplement: Multimedia Appendix 6 [file jmir_v27i1e59653_app6.docx]

**Supplementary table 6.** Intra-cluster effects in the main outcome (PSS). Intention-to-treat approach.

| Assessment (n) | PSS score, M (SD) | Pre- vs. First post-intervention | | | Pre- vs. Second post-intervention | | | First vs. Second post-intervention | | | Second vs. Third post-intervention | | | |
| --- | --- | --- | --- | --- | --- | --- | --- | --- | --- | --- | --- | --- | --- | --- |
|  |  | **B** | **t (*P*)** | **d** | **B** | **t (*P*)** | **d** | **B** | **t (*P*)** | **d** | **B** | **t (*P*)** | **d** | |
| Cluster 1 | | | | | | | | | | | | | | |
| 1 (n = 81) | 17.98 (6.35) | 3.50 | **4.18**  **(< .001)** | 0.46 | 4.68 | **5.11**  **(< .001)** | 0.68 | 1.82 | 2.28  (.03) | 0.23 | -1.29 | -1.82  (.08) | | 0.17 |
| 2 (n = 74)^a^ | 18.36 (6.00) |  |  |  |  |  |  |  |  |  |  |  |  |  |
| 3 (n = 56)^b^ | 15.64 (5.85) |  |  |  |  |  |  |  |  |  |  |  |  |  |
| 4 (n = 49)^c^ | 14.29 (6.03) |  |  |  |  |  |  |  |  |  |  |  |  |  |
| 5 (n = 53)^d^ | 15.42 (7.06) |  |  |  |  |  |  |  |  |  |  |  |  |  |
| Cluster 2 | | | | | | | | | | | | | | |
| 1 (n = 48) | 18.33 (6.02) | -0.28 | -0.31  (.76) | 0.02 | **-** | | | **-** | | | **-** | | | |
| 2 (n = 38) | 17.79 (7.05) |  |  |  |  |  |  |  |  |  |  |  |  |  |
| 3 (n = 35) | 16.14 (6.26) |  |  |  |  |  |  |  |  |  |  |  |  |  |
| 4 (n = 35)^a^ | 14.54 (7.15) |  |  |  |  |  |  |  |  |  |  |  |  |  |
| 5 (n = 26)^b^ | 14.65 (6.82) |  |  |  |  |  |  |  |  |  |  |  |  |  |
| Cluster 3 | | | | | | | | | | | | | | |
| 1 (n = 60) | 16.42 (6.13) | 2.25 | 1.82 (.08)* | 0.37 | 1.88 | 1.78 (.09) | 0.28 | -0.30 | -0.23 (.82) | 0.10 | - | | | |
| 2 (n = 44) | 16.16 (7.03) |  |  |  |  |  |  |  |  |  |  |  |  |  |
| 3 (n = 46)^a^ | 15.43 (6.41) |  |  |  |  |  |  |  |  |  |  |  |  |  |
| 4 (n = 30)^b^ | 12.93 (7.21) |  |  |  |  |  |  |  |  |  |  |  |  |  |
| 5 (n = 27)^c^ | 13.63 (6.39) |  |  |  |  |  |  |  |  |  |  |  |  |  |
| Cluster 4 | | | | | | | | | | | | | | |
| 1 (n = 75) | 15.29 (5.64) | 4.52 | 2.42 (.02)* | 0.81 | 3.80 | 2.13 (.04)* | 0.74 | -0.20 | -0.16 (.87) | 0.10 | - | | | |
| 2 (n = 55) | 16.47 (5.77) |  |  |  |  |  |  |  |  |  |  |  |  |  |
| 3 (n = 51)^a^ | 16.61 (6.69) |  |  |  |  |  |  |  |  |  |  |  |  |  |
| 4 (n = 28)^b^ | 11.68 (5.41) |  |  |  |  |  |  |  |  |  |  |  |  |  |
| 5 (n = 27)^c^ | 12.22 (5.06) |  |  |  |  |  |  |  |  |  |  |  |  |  |
| Cluster 5 | | | | | | | | | | | | | | |
| 1 (n = 57) | 16.53 (7.09) | 2.90 | 2.30 (.03)* | 0.37 | 4.60 | **4.13**  **(< .001)** | 0.62 | 2.52 | 2.37 (.02)* | 0.25 | -0.62 | -0.59 (.56)* | | 0.14 |
| 2 (n = 51)^a^ | 17.25 (7.73) |  |  |  |  |  |  |  |  |  |  |  |  |  |
| 3 (n = 42)^b^ | 14.50 (7.20) |  |  |  |  |  |  |  |  |  |  |  |  |  |
| 4 (n = 32)^c^ | 12.81 (6.54) |  |  |  |  |  |  |  |  |  |  |  |  |  |
| 5 (n = 26)^d^ | 13.69 (6.29) |  |  |  |  |  |  |  |  |  |  |  |  |  |
| Cluster 6 | | | | | | | | | | | | | | |
| 1 (n = 26) | 17.27 (6.75) | -0.30 | -0.17 (.87) | 0.42 | - | | | - | | | - | | | |
| 2 (n = 15) | 17.80 (8.14) |  |  |  |  |  |  |  |  |  |  |  |  |  |
| 3 (n = 15) | 16.40 (9.40) |  |  |  |  |  |  |  |  |  |  |  |  |  |
| 4 (n = 16)^a^ | 14.69 (7.98) |  |  |  |  |  |  |  |  |  |  |  |  |  |
| 5 (n = 11)^b^ | 11.27 (8.26) |  |  |  |  |  |  |  |  |  |  |  |  |  |

***Note:*** In **bold**, effects that remained significant (*P* < .05) after applying the Benjamini-Hochberg correction. Superscripts refer to the assessment point: ^a^ means pre-intervention, ^b^ means first post-intervention, ^c^ means second post-intervention, and ^d^ means third post-intervention. The analyses reported in this table were conducted using raw data; after conducting simple imputation (mean of nearby points), some effects were potentiated and resulted statistically significant after applying the Benjamini-Hochberg correction (*).
