# Supplementary material for: Effectiveness of a Web-Based Self-Guided Intervention (MINDxYOU) for Reducing Stress and Promoting Mental Health Among Health Professionals: Results From a Stepped-Wedge Cluster Randomized Trial
Source: J Med Internet Res. 2025 Feb 3;27:e59653. doi: 10.2196/59653 (PMC11833273; doi:10.2196/59653)
Supplement: Multimedia Appendix 7 [file jmir_v27i1e59653_app7.docx]

**Supplementary table 7.** Intra-cluster effects in the main outcome (PSS). Per-protocol approach.

| Assessment (n) | PSS score, M (SD) | Pre- vs. First post-intervention | | | Pre- vs. Second post-intervention | | | First vs. Second post-intervention | | | Second vs. Third post-intervention | | | |
| --- | --- | --- | --- | --- | --- | --- | --- | --- | --- | --- | --- | --- | --- | --- |
|  |  | **B** | **t (*P*)** | **d** | **B** | **t (*P*)** | **d** | **B** | **t (*P*)** | **d** | **B** | **t (*P*)** | **d** | |
| Cluster 1 | | | | | | | | | | | | | | |
| 1 (n = 81) | 17.98 (6.35) | 4.40 | **3.80**  **(.001)** | 0.54 | 6.06 | **6.64**  **(<.001)** | 0.77 | 1.42 | 1.42  (.17) | 0.24 | -1.63 | -2.12  (.04) | | 0.24 |
| 2 (n = 74)^a^ | 18.36 (6.00) |  |  |  |  |  |  |  |  |  |  |  |  |  |
| 3 (n = 35)^b^ | 15.17 (5.72) |  |  |  |  |  |  |  |  |  |  |  |  |  |
| 4 (n = 33)^c^ | 13.73 (6.06) |  |  |  |  |  |  |  |  |  |  |  |  |  |
| 5 (n = 34)^d^ | 15.35 (7.44) |  |  |  |  |  |  |  |  |  |  |  |  |  |
| Cluster 2 | | | | | | | | | | | | | | |
| 1 (n = 48) | 18.33 (6.02) | -0.69 | -0.61  (.55) | 0.01 | **-** | | | **-** | | | **-** | | | |
| 2 (n = 38) | 17.79 (7.05) |  |  |  |  |  |  |  |  |  |  |  |  |  |
| 3 (n = 35) | 16.14 (6.26) |  |  |  |  |  |  |  |  |  |  |  |  |  |
| 4 (n = 35)^a^ | 14.54 (7.15) |  |  |  |  |  |  |  |  |  |  |  |  |  |
| 5 (n = 16)^b^ | 14.44 (6.43) |  |  |  |  |  |  |  |  |  |  |  |  |  |
| Cluster 3 | | | | | | | | | | | | | | |
| 1 (n = 60) | 16.42 (6.13) | 2.41 | 1.94 (.07) | 0.56 | 1.44 | 1.05 (.31) | 0.36 | -1.06 | -0.89 (.39) | 0.21 | - | | | |
| 2 (n = 44) | 16.16 (7.03) |  |  |  |  |  |  |  |  |  |  |  |  |  |
| 3 (n = 46)^a^ | 15.43 (6.41) |  |  |  |  |  |  |  |  |  |  |  |  |  |
| 4 (n = 17)^b^ | 11.71 (6.85) |  |  |  |  |  |  |  |  |  |  |  |  |  |
| 5 (n = 17)^c^ | 13.12 (6.59) |  |  |  |  |  |  |  |  |  |  |  |  |  |
| Cluster 4 | | | | | | | | | | | | | | |
| 1 (n = 75) | 15.29 (5.64) | 2.44 | 0.79 (.45) | 0.82 | 2.08 | 0.86 (.41) | 0.60 | -0.78 | -0.48 (.64) | 0.17 | - | | | |
| 2 (n = 55) | 16.47 (5.77) |  |  |  |  |  |  |  |  |  |  |  |  |  |
| 3 (n = 51)^a^ | 16.61 (6.69) |  |  |  |  |  |  |  |  |  |  |  |  |  |
| 4 (n = 9)^b^ | 12.22 (3.53) |  |  |  |  |  |  |  |  |  |  |  |  |  |
| 5 (n = 12)^c^ | 13.00 (5.33) |  |  |  |  |  |  |  |  |  |  |  |  |  |
| Cluster 5 | | | | | | | | | | | | | | |
| 1 (n = 57) | 16.53 (7.09) | 3.00 | 1.83 (.08) | 0.62 | 5.19 | **3.89**  **(.001)** | 0.69 | 1.00 | 0.83 (.42) | 0.12 | -0.65 | -0.52 (.61) | | 0.11 |
| 2 (n = 51)^a^ | 17.25 (7.73) |  |  |  |  |  |  |  |  |  |  |  |  |  |
| 3 (n = 23)^b^ | 13.04 (5.76) |  |  |  |  |  |  |  |  |  |  |  |  |  |
| 4 (n = 22)^c^ | 12.32 (6.40) |  |  |  |  |  |  |  |  |  |  |  |  |  |
| 5 (n = 20)^d^ | 13.05 (5.93) |  |  |  |  |  |  |  |  |  |  |  |  |  |
| Cluster 6 | | | | | | | | | | | | | | |
| 1 (n = 26) | 17.27 (6.75) | 6.00 | 3.00 (.21) | 0.44 | - | | | - | | | - | | | |
| 2 (n = 15) | 17.80 (8.14) |  |  |  |  |  |  |  |  |  |  |  |  |  |
| 3 (n = 15) | 16.40 (9.40) |  |  |  |  |  |  |  |  |  |  |  |  |  |
| 4 (n = 16)^a^ | 14.69 (7.98) |  |  |  |  |  |  |  |  |  |  |  |  |  |
| 5 (n = 3)^b^ | 10.33 (11.37) |  |  |  |  |  |  |  |  |  |  |  |  |  |

***Note:*** In **bold**, effects that remained significant (*P* < .05) after applying the Benjamini-Hochberg correction. Superscripts refer to the assessment point: ^a^ means pre-intervention, ^b^ means first post-intervention, ^c^ means second post-intervention, and ^d^ means third post-intervention. The analyses reported in this table were conducted using raw data; after conducting simple imputation (mean of nearby points), no effects were potentiated and resulted statistically significant after applying the Benjamini-Hochberg correction.
